# Evaluation of the effectiveness of caudal epidural steroid injection as an adjuvant to ganglion impar pulsed radiofrequency therapy in chronic coccygodynia

**DOI:** 10.1016/j.heliyon.2024.e31161

**Published:** 2024-05-11

**Authors:** Gülçin Gazioğlu Türkyılmaz, Şebnem Rumeli

**Affiliations:** a Pain Clinic, Ministry of Health Bursa City Hospital, Bursa, Turkey; b Division of Pain Medicine, Department of Anesthesiology and Reanimation, Faculty of Medicine, Mersin University, Mersin, Turkey

**Keywords:** Chronic pain, Coccygodynia, Ganglion impar pulse radiofrequency treatment, Caudal epidural steroid injection

## Abstract

**Background:**

This study aimed to evaluate the effectiveness of adding caudal epidural steroid injection (CESI) to ganglion impar pulsed radiofrequency (GI-PRF) therapy in patients with refractory chronic coccygodynia, and to determine the effect of pain duration and trauma on treatment success.

**Materials and methods:**

Forty patients who underwent GI-PRF (n = 20) or GI-PRF + CESI (n = 20) were retrospectively assessed for age, gender, pain duration, history of trauma to the coccyx, Numerical Rating Scale (NRS) pain scores pre-procedure and 1, 3, and 6 months post-procedure and satisfaction rates at 6 months post-procedure. Satisfaction was categorized as excellent, high, moderate, and low.

**Results:**

In both groups, NRS scores were significantly decreased at 6 months (pre-vs. 6 mo. post-procedure: GI-PRF [8 vs. 5], GI-PRF + CESI [8.5 vs. 3.5]; p < 0.001). The proportion of patients reporting excellent satisfaction was significantly higher in the GI PRF + CESI group (50 % vs. 15 %; p < 0.05). Patients with trauma history in the GI-PRF + CESI group had significantly lower median NRS values at 6 months compared to patients in both groups with negative trauma history (p < 0.02). Within the GI-PRF only group, patients with trauma history had significantly lower NRS scores at 6 months than those without a history of trauma (p = 0.012). Pain duration did not significantly impact satisfaction levels (p = 0.055).

**Conclusion:**

GI-PRF therapy was effective in coccygodynia, especially in patients with positive trauma history, and adjuvant CESI increased patient satisfaction by providing better pain control.

## 1 Introduction

Coccygodynia (or coccydynia) refers to pain in the region of the coccyx, the lowest part of the spine. The most common etiology is trauma, which may occur as a result of falls, difficult or instrumented birth, or prolonged sitting on hard and uncomfortable surfaces. Non-traumatic causes include degenerative disc disease, sacrococcygeal joint hypermobility, neoplasms, and psychological causes. The prevalence of coccygodynia is 5 times higher in women than men [1]. The most common symptom is midline pain below the sacrum and above the anus. Symptoms are usually exacerbated when sitting or rising from a seated position and significantly reduce patients’ quality of life [2].

First-line treatment options include the use of pressure-reducing cushions, nonsteroidal anti-inflammatory drugs, levator ani relaxation exercises, transcutaneous electrical nerve stimulation and manual manipulation of the coccyx [1]. Interventional treatment modalities such as caudal epidural steroid injection (CESI), ganglion impar block (GIB), radiofrequency (RF) ablation and chemical neurolysis can be applied if pain is refractory to these treatments [3].

In the treatment of chronic coccygodynia, ganglion impar pulse radiofrequency (GI-PRF) therapy has been shown to provide significantly longer-lasting pain relief than GIB [3]. In pulse radiofrequency (PRF), the RF current is applied in a pulsed manner with low electrode tip temperatures. It has been suggested that the mechanism underlying the neuromodulatory effects of PRF arises from the electromagnetic field it produces and is related to increased activity of the noradrenergic and serotonergic descending pathways [4,5]. In another study, both GIB and CESI were shown to be useful treatment methods in coccygodynia nonresponsive to conservative treatments [6]. In our review of the literature, we found no study comparing the effectiveness of GI-PRF therapy with and without adjuvant CESI in chronic coccygodynia. The primary aim of this study was to evaluate the effectiveness of adding CESI to GI-PRF therapy in patients with chronic coccygodynia refractory to conservative treatment. Our secondary aim was to determine whether pain duration and trauma history have an effect on treatment success.

## 2 Materials and Methods

### 2.1 Study design

This was a retrospective study.

### 2.2 Setting

The study was approved by the Clinical Research Ethics Committee of Bursa City Hospital (date: 13.09.2023, decision number 2023-15/1). The records of patients who presented to the algology outpatient clinic with complaints of coccyx pain and were diagnosed with coccygodynia between November 1, 2021 and June 30, 2023 were retrospectively reviewed. Those who had pain for at least 3 months, did not benefit from any conservative treatments, had no infection or bleeding disorder and underwent GI-PRF alone or GI-PRF with concurrent CESI in the same session were included in the study. Patients with less than 3 months of pain, no previous conservative treatment, local or systemic infection, bleeding disorder, allergy to local anesthetics and/or contrast agents, failure to give written and verbal consent for interventional procedures, additional pathology such as mass or disc herniation on pre-procedural lumbosacral MRI (magnetic resonance imaging) and missing follow-up data were excluded.

Patients' age, gender, duration of pain, history of trauma to the coccyx, and pain intensity assessed using the Numerical Rating Scale (NRS) were recorded at initial presentation to the outpatient clinic and 1, 3, and 6 months after the procedure. The NRS is a segmented numeric version of the visual analog scale (VAS) in which a respondent selects a whole number (0–10 integers) that best reflects the intensity of their pain. An 11-point numeric scale (NRS 11) with 0 representing one pain extreme (e.g., “no pain”) and 10 representing the other pain extreme (e.g.,“pain as bad as you can imagine” and “worst pain imaginable”) [7]. NRS values of all patients undergoing interventional pain procedures in our clinic are recorded before the procedure and at routine outpatient visits 1 and 3 months after the procedure. For this study, pain assessments of patients at 6 months postprocedure were performed with both NRS values and patient satisfaction. Patient satisfaction was categorized as “excellent” (≥75 % pain reduction), “high” (50%–74 % pain reduction), “moderate” (25%–49 % pain reduction) and “low” (<25 % reduction or increase in pain) [8]. For this study, patients were contacted at 6 months post-procedure and questioned about the percentage of pain reduction compared to pre-procedure and NRS values at 6 months post-procedure.

### 2.3 Procedures

All patients included in the study were evaluated by a single algologist, and the GI-PRF and CESI procedures were performed by the same physician (G.T.).

**Fig. 1:**
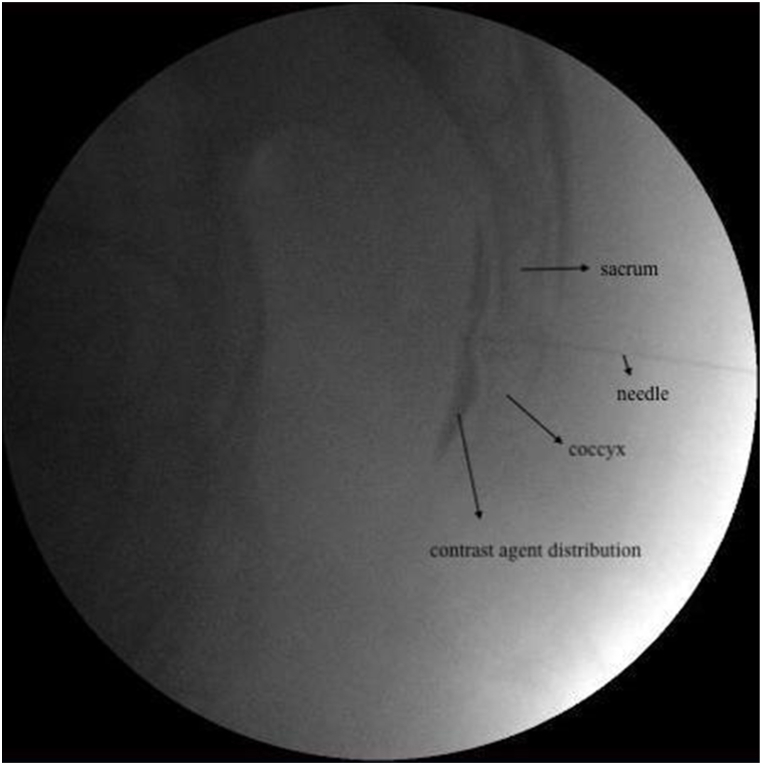
Ganglion impar pulsed radiofrequency (GI-PRF) lateral image.

**Ganglion Impar Pulsed Radiofrequency Therapy:** The procedures were performed under sterile conditions, with standard monitorization and under fluoroscopic guidance. After mild sedation, the patients were placed in prone position and asepsis of the intergluteal region was performed using 10 % povidone-iodine. The needle insertion site was determined as the sacrococcygeal or first coccygeal joint, which was best visualized in the lateral fluoroscopic view. After subcutaneous infiltration of 1 mL 2 % lidocaine, a 10-cm 22-gauge (G) RF needle with 10-mm active tip (Top-JPN) was inserted through the joint until the needle tip was observed in front of the coccyx. A contrast agent (Omnipaque 300, GE Healthcare, Ireland) was administered to confirm correct needle localization (Fig. 1). After properly positioning the RF needle, a radiofrequency device (TOP-TLG 10 STP) was used to ensure that impedance was <500 Ω at the PRF generator. Application of sensory stimulation at 50 Hz induced paresthesia in the sacrococcygeal region at < 1 V. No distal muscle contraction was observed with 2 Hz motor stimulation. After observing the appropriate responses, PRF therapy was applied at 45 V and 42° for 240 s. No drug was injected during the GI-PRF procedure. Any adverse events and complications were recorded in the patients’ follow-up records.

**Fig. 2:**
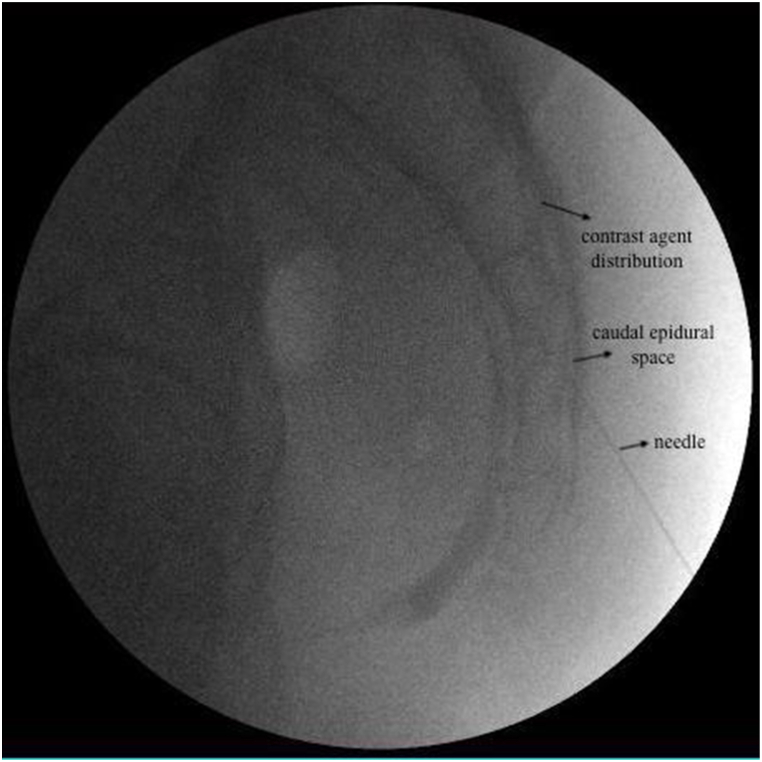
Caudal epidural steroid injection (CESI) lateral image.

**Caudal Epidural Steroid Injection:** Under fluoroscopic guidance, the caudal epidural space was visualized in the lateral view. After cutaneous infiltration of 1 mL 2 % lidocaine, a 22-G spinal needle was inserted into the caudal epidural space after confirmation of correct localization by contrast agent administration (Fig. 2). A total of 6 mL of fluid was administered: 80 mg/2 mL methyl prednisolone acetate and 4 mL 0.9 % NaCl.

All patients were advised to use appropriate coccyx cushion after the procedure.

## 3 Statistical analysis

Continuous variables were tested for normal distribution using the Shapiro-Wilk test. Due to the non-normal distribution of age, duration of pain, and NRS scores, descriptive statistics are reported as median, minimum, and maximum values, and comparisons between groups were conducted using the Mann-Whitney *U* test and Kruskal-Wallis test. For categorical variables such as gender, history of trauma, and procedure satisfaction, comparisons between groups were performed using chi-square and Fisher-Freeman-Halton tests. In subgroup analyses addressing procedure satisfaction, the Bonferroni correction was applied to assess the significance of p-values. Within-group comparisons were made by using the Wilcoxon signed rank test. Data analyses were conducted using SPSS (IBM SPSS Statistics for Windows, Version 23.0; IBM Corp., Armonk, NY), and the type I error rate was set at 5 % for statistical comparisons.

## 4 Results

Of 42 patients who presented to our clinic with complaints of chronic coccygodynia during the study period, 2 were excluded from the study due to missing follow-up data. Therefore, the study included 40 patients who underwent GI-PRF alone or GI-PRF + CESI for pain treatment. The mean age of the patients in the study was 45.7 ± 9.6 years, 77.5 % (n = 31) were female, and 55 % (n = 22) had a history of trauma. None of the patients experienced complications during or after the procedure.

There was no significant difference between the two groups in terms of age, gender, duration of pain at the time of presentation to the outpatient clinic, or history of trauma (p > 0.05) (Table 1).

**Table 1:** Comparison of demographic characteristics, pain duration, trauma history, satisfaction and Numerical Rating Scale (NRS) between the study groups.

	GI-PRF (n = 20)	GI-PRF + CESI (n = 20)	p value
**Age (yr)**	50 (31–57)	45 (21–66)	0.253^a^
**Gender**			
Female, n (%)	16 (80)	13 (65)	0.288^b^
Male, n (%)	4 (20)	7 (35)	
**Duration of Pain (mo)**	14 (3–96)	24 (4–96)	0.989^a^
**Trauma History, n (%)**	11(55)	11 (55)	>0.999^b^
**Satisfaction, n (%)**			
Excellent	3 (15)	10 (50)	**0.019**^c^
High	7 (35)	2 (1)	
Moderate	0	2 (10)	
Low	10 (50)	6 (30)	
**NRS score**			
Pre-procedure	8 (7–9)^d^	8.5 (7–9)^d^	0.862
1 month post-procedure	4 (1–8)	2 (1–8)	0.060
3 months post-procedure	4 (1–8)	2 (1–8)	0.102
6 months post-procedure	5 (1–8)	3.5 (1–8)	0.121

GI-PRF: Ganglion impar pulsed radiofrequency, CESI: Caudal epidural steroid injection.

Continuous data shown as median (range).

a Mann-Whitney *U* test.

b Chi-square test.

c Fisher-Freeman-Halton test.

d (p < 0.001).

### 4.1 NRS

In both groups, NRS scores were significantly lower at 1, 3, and 6 months after the procedure compared to before the procedure (p < 0.001 for all) (Table 1). There was no significant difference between the groups in terms of NRS scores before or after treatment (p > 0.05).

### 4.2 Patient satisfaction

At 6 months after the procedure, there was a significant difference in patient satisfaction between the groups (p = 0.019). The proportion of patients reporting excellent satisfaction with the procedure was higher in the GI-PRF + CESI group compared to the GI-PRF only group (50 % vs. 15 %; p < 0.05). The rates of patients with high, moderate, and low satisfaction did not differ between the study groups (p > 0.05) (Table 1).

### 4.3 Trauma effects

NRS scores at all time points differed significantly between patients with and without a history of trauma (p < 0.05). In the subgroup analyses, median NRS scores at 1, 3, and 6 months after the procedure were significantly lower in patients with trauma history who underwent GI-PRF and CESI compared to those in both the GI-PRF and GI PRF + CESI groups who did not have a history of trauma (p < 0.02). Among the patients who underwent GI-PRF only, median NRS scores at 6 months were significantly lower in those with a history of trauma compared to those without (p = 0.012) (Table 2).

**Table 2:** Comparison of NRS scores, satisfaction levels and pain durations between patients with and without a history of coccyx trauma.

	Positive Trauma History		Negative Trauma History		
	GI-PRF (n = 11)	GI-PRF + CESI (n = 11)	GI-PRF (n = 9)	GI-PRF + CESI (n = 9)	p-value^a^^”^
**NRS score**					
Pre-procedure	9 (8–9)	9 (8–9)	8 (7–9)	8 (7–9)	**0.006**
1 month post-procedure	4 (1–8)	2 (1–4)	7 (4–8)	7 (1–8)	**<0.001**
3 months post-procedure	4 (1–8)	2 (1–4)	7 (4–8)	7 (1–8)	**<0.001**
6 months post-procedure	4 (1–8)	2 (1–4)	8 (6–8)	7 (1–8)	**<0.001**
**Satisfaction Level**					
Excellent	3^Ɣ,¥,Ŧ^(27.3 %)	9^¥^ (81.8 %)	0^Ŧ^	1^Ɣ,Ŧ^(11.1 %)	
High	7^Ɣ^ (63.6 %)	1^¥^ (9.1 %)	0^¥^	1^Ɣ,¥^ (11.1 %)	
Moderate	0^Ɣ^	1^Ɣ^ (9.1 %)	0^Ɣ^	1^Ɣ^ (11.1 %)	**<0.001**^b^
Low	1^Ɣ^ (9.1 %)	0^Ɣ^	9^¥^ (100 %)	6^¥^ (66.7 %)	
**Duration of pain (mo)**	8 (3–96)	60 (12–96)	24 (4–60)	24 (9–96)	**0.038**^a^

GI-PRF: Ganglion impar pulsed radiofrequency, CESI: Caudal epidural steroid injection.

Data are given as median (range) and n (%).

Each superscript symbol denotes a subset of column categories whose column proportions do not differ significantly (p > 0.05).

a Kruskal-Wallis test.

b Fisher-Freeman-Halton test.

In subgroup analyses of satisfaction, the proportion of patients reporting excellent satisfaction with the procedure at 6-month follow-up was significantly higher among patients in the GI-PRF + CESI group with a history of coccyx trauma when compared with the other groups (p < 0.05) (Table 2).

### 4.4 Pain duration

Although pain duration differed between the groups (p = 0.038), no significant difference was found in pairwise comparisons (Table 2). There was no difference between the groups in terms of satisfaction levels according to pain duration (p = 0.055) (Table 3).

**Table 3:** Evaluation of pain duration according to procedure satisfaction in the study groups.

Satisfaction Level	n	GI-PRF Pain duration (mo)	n	GI-PRF + CESI Pain duration (mo)
**Excellent**	3^a^	9 (8–9)	10	9 (8–9)
**High**	7	4 (1–8)	2^a^	2 (1–4)
**Moderate**	-a	4 (1–8)	2^a^	2 (1–4)
**Low**	10	4 (1–8)	6	2 (1–4)
**p value**	0.055^b^			

GI-PRF: Ganglion impar pulsed radiofrequency, CESI: Caudal epidural steroid injection.

Data are given as median (range).

a Excluded from the analysis because the number of data was insufficient for statistical comparison.

b Kruskal-Wallis test.

## 5 Discussion

Our study is the first to evaluate the effectiveness of adding CESI to GI-PRF therapy in patients with chronic coccygodynia refractory to conservative treatment. Treatment was beneficial in both groups treated with GI PRF and GI PRF + CESI. However, better results were obtained in the CESI + GI PRF group compared to the other group in terms patient satisfaction. Patients with a history of trauma to the coccyx benefited more from both treatments than patients without a history of trauma.

Coccygodynia is five times more common in women than in men [1,9]. Although the average age at onset is 40, it can be seen in a wide age group [10]. The results of our study were also consistent with the literature.

PRF procedures main advantage is that it provides long-term pain control without complications [11]. There are very few studies evaluating the efficacy of GI PRF in the treatment of chronic coccygodynia. In a study comparing the efficacy of GIB and GI-PRF therapy in the treatment of chronic coccygodynia, it was found that NRS scores at 6 months were significantly lower and patient satisfaction was significantly higher in the GI-PRF group [3]. In our study, all patients who underwent GI-PRF reported a significant improvement in pain for 6 months, in accordance with the literature.

There are a limited number of studies investigating the effect of CESI on coccygodynia. CESI can be used to treat lumbar pathologies such as disc herniation and spinal stenosis, as well as lower sacral radicular pain, including coccydynia [12]. A retrospective study comparing the effectiveness of transrectal manipulation with GIB and CESI in coccygodynia indicated that manipulation with GIB was more effective in improving pain and pain-free sitting time at 6-month follow-up [13]. In another randomized, prospective study comparing the efficacy of GIB and CESI in coccygodynia, a significant improvement in pain was observed in the GIB group compared to the CESI group at 3 weeks, but at 3 months this difference disappeared and both groups showed a significant decrease in pain compared to baseline [4]. The authors attributed the effectiveness of CESI in reducing coccygodynia to its relief of pain associated with lumbar pathology and blocking of the sacrococcygeal nerves, which are an important source of pain in coccygodynia [4]. Corticosteroid injection has also been recommended in the literature based on the presumption that the cause of coccygodynia is inflammatory [14]. In our study, although the addition of CESI to GI-PRF therapy enhanced pain reduction at 6-month follow-up, the difference was not statistically significant. However, it was also observed that patient satisfaction was higher at 6 months after the procedure in the group who received CESI. This may be a result of the blocking effect of the corticosteroid on the sacral plexus and coccygeal plexus, which contain the pudendal and cutaneous nerves of the inferior gluteal region, as well its anti-inflammatory effect in the region.

The most common cause of coccydynia is a direct fall on the coccyx or recurrent microtrauma [15]. In our study, 55 % of the patients had a history of trauma. In a study involving 748 patients with coccygodynia who underwent local injection, manipulation under anesthesia, or coccygectomy and were followed up for at least 6 months, it was concluded that patients with coccyx trauma responded better to the treatment methods applied [16]. In a retrospective evaluation of patients who underwent coccygectomy after not responding to any conservative treatment methods, it was determined that 88 % of patients with trauma-related pain and 38 % of patients with idiopathic coccygodynia benefited significantly from the surgery [17]. In another retrospective study evaluating the efficacy of GI-PRF in coccygodynia, the etiology was trauma in 80 % (21/26) of the patients and 80 % (17/21) of those patients were in the treatment success group, defined as those who showed at least 50 % reduction in pain at 3 months [18]. In keeping with the literature, among the patients in our study who underwent GI-PRF, those with a history of trauma reported significantly greater decrease in pain at 6 months after the procedure. Prospective and comprehensive studies are needed to obtain more definitive results.

Chronic pain may cause irreversible changes in the brain that can adversely affect various aspects of patient well-being, including cognitive and mental health and overall quality of life, potentially making treatment even more difficult over time [19,20]. In a retrospective study of 102 patients with chronic coccygodynia examining the factors affecting treatment success at 3 months after GIB, a symptom duration longer than 24.5 months was associated with significantly lower treatment success [21]. Our literature search yielded no previous study examining the relationship between pain duration and the effectiveness of GI-PRF and CESI therapy. In our study, the duration of pain in the GI-PRF and GI-PRF + CESI groups (median 14 and 24 months, respectively) had no impact on satisfaction levels at 6 months after the procedure. Conducting studies with larger patient samples may provide more definitive results.

Limitations of this study are its small sample, retrospective design, and that radiological fracture, subluxation, or curvature classifications were not made for patients with a history of trauma to the coccyx. In addition, pre- and post-procedural dose changes of analgesic medications used by the patients could have been recorded, non-traumatic causes of coccygodynia such as history of lumbosacral surgery and psychiatric disorders could have been questioned and their impact on procedural success could have been evaluated.

However, we think that this study will guide more comprehensive prospective studies including radiological classifications after coccygeal trauma.

GI-PRF therapy was found to be effective in coccygodynia, especially in patients with a history of trauma, and adjuvant CESI further increased patient satisfaction by providing better pain control. Long-term follow-up studies with larger patient groups are needed to more clearly demonstrate the effectiveness of GI-PRF therapy with adjuvant CESI in chronic coccygodynia patients with trauma history.

## Ethics statement

Patients gave informed consent for the publication of all their data images.

## Funding

No funding to declare.

## Data availability statement

All data analyzed in this study are included in the published article. The data that support the findings of this study are available from the corresponding author upon reasonable request.

## Financial disclosure

There is no financial disclosure

## Ethical approval

Approval was obtained from Bursa City Hospital Clinical Research Ethics Committee with the decision dated 13.9.2023 and numbered 2023-15/1.

## CRediT authorship contribution statement

**Gülçin Gazioğlu Türkyılmaz:** Writing – original draft, Visualization, Validation, Software, Resources, Project administration, Methodology, Investigation, Funding acquisition, Data curation, Conceptualization. **Şebnem Rumeli:** Writing – review & editing, Supervision, Formal analysis.

## Declaration of competing interest

The authors declare that they have no known competing financial interests or personal relationships that could have appeared to influence the work reported in this paper.
