# Supplementary material for: Evaluation of the effectiveness of caudal epidural steroid injection as an adjuvant to ganglion impar pulsed radiofrequency therapy in chronic coccygodynia
Source: Heliyon. 2024 May 11;10(10):e31161. doi: 10.1016/j.heliyon.2024.e31161 (PMC11109874; doi:10.1016/j.heliyon.2024.e31161)
Supplement: Multimedia component 1 [file mmc1.pdf]

EDITORIAL CERTIFICATE

This certificate confirms that the accompanying document was edited for proper English language, grammar, punctuation, spelling, and overall style by the following highly qualified native speaker:

Jacqueline Renee Gutenkunst
Scientific/Medical Editor
Baltimore, MD

Manuscript Title

Evaluation of the Effectiveness of Caudal Epidural Steroid Injection as an Adjuvant to Ganglion Impar Pulsed Radiofrequency Therapy in Chronic Coccygodynia

Date Issued

28 January 2024

Certificate Number

3bgZyb2mTQ

Documents receiving this certification are English-ready for publication, pending acceptance of all changes and suggestions. To verify this certificate, please contact: jackigutenkunst@gmail.com.
